# A Novel Variant in *NR5A1* Resulting in Normosmic Hypogonadotropic Hypogonadism With Short Stature

**DOI:** 10.1210/jcemcr/luaf213

**Published:** 2025-10-07

**Authors:** Sapan Shah, Soumik Goswami, Nilanjan Sengupta, Arjun Baidya, Roohi Nanda, Rajdeep Basu

**Affiliations:** Department of Endocrinology, Nil Ratan Sircar Medical College and Hospital, Nil Ratan Sircar Medical College, Kolkata, West Bengal 700014, India; Department of Endocrinology, Nil Ratan Sircar Medical College and Hospital, Nil Ratan Sircar Medical College, Kolkata, West Bengal 700014, India; Department of Endocrinology, Nil Ratan Sircar Medical College and Hospital, Nil Ratan Sircar Medical College, Kolkata, West Bengal 700014, India; Department of Endocrinology, Nil Ratan Sircar Medical College and Hospital, Nil Ratan Sircar Medical College, Kolkata, West Bengal 700014, India; Department of Endocrinology, Nil Ratan Sircar Medical College and Hospital, Nil Ratan Sircar Medical College, Kolkata, West Bengal 700014, India; Department of Endocrinology, Nil Ratan Sircar Medical College and Hospital, Nil Ratan Sircar Medical College, Kolkata, West Bengal 700014, India

**Keywords:** *NR5A1*, delayed puberty, normosmic hypogonadotropic hypogonadism

## Abstract

We report a case of a 16-year-old male with normosmic hypogonadotropic hypogonadism and short stature, harboring a homozygous variant in the *NR5A1* gene. The patient presented at 16 years of age with poor height and weight gain for the last 8 years along with delayed puberty and absence of secondary sexual characteristics. On examination, he had severe short stature, unambiguous external genitalia, micropenis, and prepubertal sexual maturation. Investigations revealed inappropriately normal gonadotropins with low testosterone, while other hormonal assessments were normal, including age- and sex-matched IGF-1. Magnetic resonance imaging of the hypothalamic-pituitary axis along with the olfactory system was normal. Whole-exome sequencing revealed a homozygous variant in the *NR5A1* gene. This case suggests that *NR5A1* can be associated with isolated congenital hypogonadotropic hypogonadism.

## Introduction

Congenital hypogonadotropic hypogonadism (CHH) is a rare genetic disorder that results from either defective secretion or action of GnRH. Clinically, it is manifested by incomplete or absent pubertal development and infertility. Biochemically, it is defined as a low sex steroid level (testosterone and estradiol) and low or inappropriately normal FSH and LH [1]. Other hormonal axes remain normal, and there is an absence of any hypothalamic-pituitary structural lesion on magnetic resonance imaging. In males, CHH presents as micropenis and/or cryptorchidism in childhood. During adolescence, it presents with a lack of virilization and delayed puberty (that is, testicular volume <4 mL beyond 14 years of age). In females, it is manifested by primary amenorrhea and the absence of breast development. Additionally, CHH may present with various nonreproductive phenotypes. Anosmia or hyposmia is associated with CHH in some patients and is termed Kallmann syndrome. Kallmann syndrome may be associated with other phenotypic abnormalities like synkinesia (mirror movements), cleft lip/palate, dental agenesis, unilateral renal agenesis, and skeletal abnormalities [2]. Others present with a normal sense of smell without any other anomaly, termed normosmic isolated hypogonadotropic hypogonadism (nIHH). Eunuchoid body proportions (arm span >5 cm of height and low upper to lower body segment ratio) are present because of a lack of epiphyseal fusion due to low estradiol levels. CHH is characterized by vast genetic heterogeneity, with mutations in >30 genes reported to date, some of which have been implicated in both Kallmann syndrome and nIHH [3].

*NR5A1,* also known as steroidogenic factor 1 (*SF1*), is an orphan nuclear receptor that regulates many genes involved in gonadal development, sexual differentiation, steroidogenesis, and reproduction. A broad spectrum of phenotypes has been reported with *NR5A1* variants, ranging from 46,XY disorders of sex development with or without primary adrenal insufficiency to primary ovarian insufficiency in 46,XX individuals [4]. Also, the role of *NR5A1* in the development of the hypothalamic-pituitary-gonadal axis and regulation of gonadotropin production in mouse models has been reported in 1 study [5].

Here, we report a case of a 16-year-old male with nIHH and short stature without any adrenal insufficiency carrying a novel homozygous variant of the *NR5A1* gene.

## Case Presentation

A 16-year-old boy presented to us with concerns of poor height and weight gain in comparison to his peers since the age of 8 years, which was noticed by the patient himself and also by his parents. He was also concerned about the lack of penile growth and other secondary sexual characteristics. The parents were concerned about the small penile length since birth. However, previous documentation regarding penile length was not available. There was no history of headache, visual disturbance, anosmia, chronic systemic illness, chronic diarrhea, abdominal pain, or sensitivity to gluten-containing food. He was born prematurely at 32 weeks of gestation by normal vaginal delivery and was kept in the neonatal intensive care unit for 2 days. There was no history of cryptorchidism at birth nor of hypoglycemia or prolonged jaundice. All developmental milestones were attained on time. There was no history of delayed puberty or infertility in parents or extended family.

## Diagnostic Assessment

**Figure 1.**
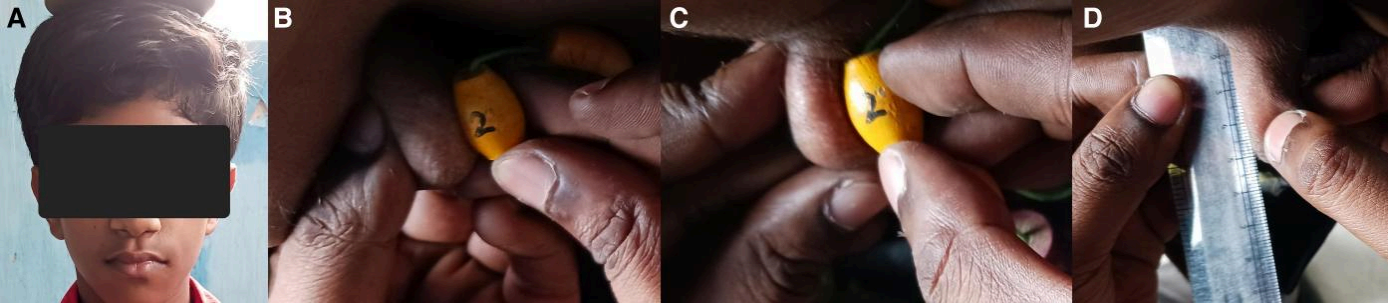
Clinical features of the index case. (A) Absence of facial hair. (B- C) Testicular volume 2 mL bilaterally measured by Prader orchidometer. (D) Stretched penile length is 5 cm.

**Figure 2.**
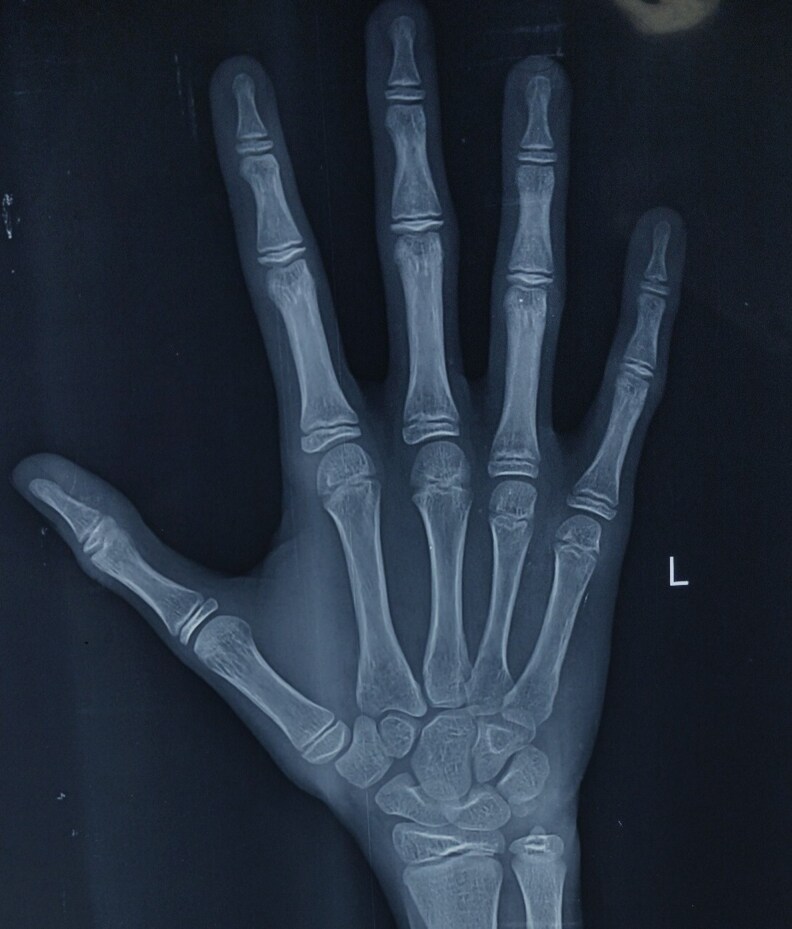
X-ray of left hand and wrist is suggestive of a bone age of approximately 11 to 12 years according to the Greulich and Pyle bone age estimation atlas.

On examination, his height was 138 cm (height SD score was −3.61). His mother's height was 150 cm, his father's height was 161 cm, and midparental height and target height were 162 cm and 164.2 cm, respectively. Target height SD score was −1.21. His weight was 29 kg, and his body mass index was 15.23 kg/m^2^ (between the third to fifth percentile). The arm span was 144 cm (>5 cm above height), and the upper segment and lower segment ratio was 0.77. Facial hair was absent. (Fig. 1A). Both the testes were in the scrotum with a volume of 2 mL for each (Fig. 1B and 1C); stretched penile length was 5 cm (Fig. 1D). His sexual maturity rating was prepubertal according to the Tanner sexual maturity rating. Synkinetic movements were absent on examination. There was no midline defect or bony abnormality. Bone age was between 11 and 12 years as per the Greulich and Pyle hand and wrist bone age estimation atlas (Fig. 2).

His blood investigations were significant for low serum testosterone with low-normal LH. Other parameters were within normal limits. A summary of laboratory findings is presented in Table 1.

**Table 1.** Investigations

Investigations	Patient's value	Reference range
Hemoglobin	11.8 g/dL	13-16 g/dL
Serum creatinine	0.9 mg/dL	0.5-1 mg/dL
	(79.58 µmol/L)	(44.21-88.42 µmol/L)
SGPT	24 U/L	<45 U/L
SGOT	23 U/L	<35 U/L
ALP	179 U/L	54-369 U/L
Anti-tissue transglutaminase IgA (anti-tTG IgA)	3.26 U/mL	<10 U/mL
Total serum IgA	301 mg/dL	61-356 mg/dL
IGF-1	479 ng/mL	160-480 ng/mL (age and sex specific)
	(62.62 nmol/L)	(20.91-62.75 nmol/L)
TSH	1.5 µIU/mL	0.35-4.6 µIU/mL
	(1.5 mIU/L)	(0.35-4.6 mIU/L)
Free T4	1.0 ng/dL	0.89-1.8 ng/dL
	(12.87 nmol/L)	(11.45-23.16 nmol/L)
Prolactin	4.3 ng/mL	1.5-14.7 ng/mL
FSH	5.0 mIU/mL	0.7-11.1 mIU/mL
	(5 IU/L)	(0.7-11.1 IU/L)
LH	1.1 mIU/mL	0.8-7.6 mIU/mL
	(1.1 IU/L)	(0.8-7.6 IU/L)
Total testosterone	0.77 ng/mL	1.58-8.26 ng/mL
	(2.66 nmol/L)	(5.47-28.63 nmol/L)
8 Am serum cortisol	15.4 µg/dL	5-25 µg/dL
	(42.48 nmol/L)	(13.79-68.96)
Plasma ACTH	17.28 pg/mL	6.17-58.2 pg/mL
	(3.80 pmol/L)	(1.35-12.81 pmol/L)

Values in parentheses are International System of Units.

Abbreviations: ACTH, adrenocorticotropic hormone; ALP, alkaline phosphatase; FSH, follicle-stimulating hormone; IGF-1, insulin-like growth factor-1; LH, luteinizing hormone; SGOT, serum glutamic-oxaloacetic transaminase; SGPT, serum glutamic pyruvic transaminase; TSH, thyroid-stimulating hormone.

**Figure 3.**
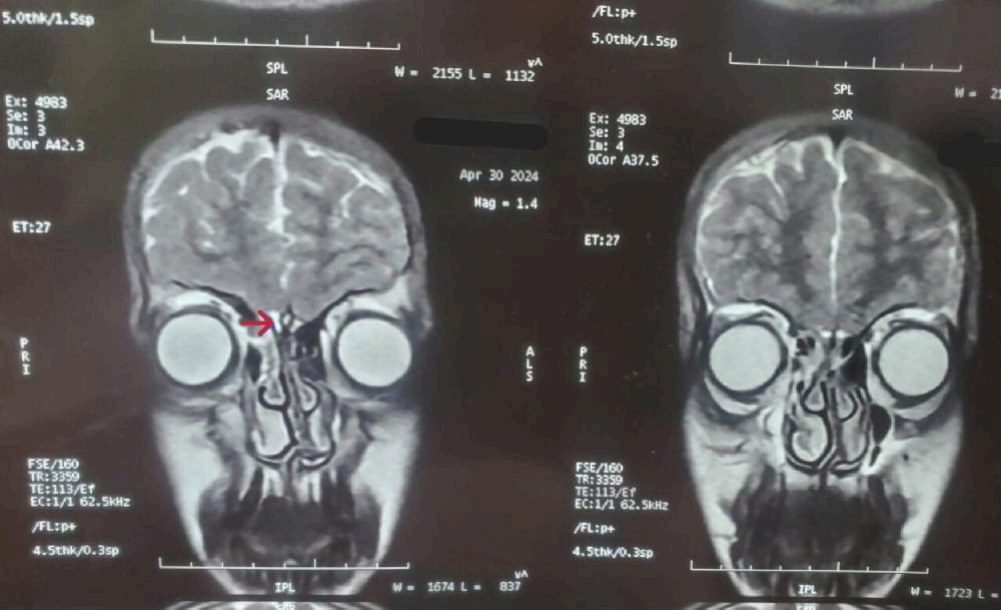
Coronal section of magnetic resonance imaging brain shows normal olfactory bulb (red arrowhead), which is bilateral symmetrical.

Magnetic resonance imaging did not reveal any abnormality in the hypothalamic-pituitary region, and the olfactory tract and bulb were normal bilaterally (Fig. 3).

Whole-exome sequencing identified a homozygous deletion (c. 1379delA) in exon 7 of the *NR5A1* gene, which can lead to frameshift and consequent elongation of the resultant protein. (p. Gln460ArgfsTer36). This variant has been interpreted as a variant of uncertain significance. Parental genetic testing could not be performed due to limited financial resources.

## Treatment

Fifty mg intramuscular injectable testosterone was initiated at monthly intervals as initial management. The dose of testosterone was escalated gradually at 6-month intervals.

## Outcome and Follow-up

**Figure 4.**
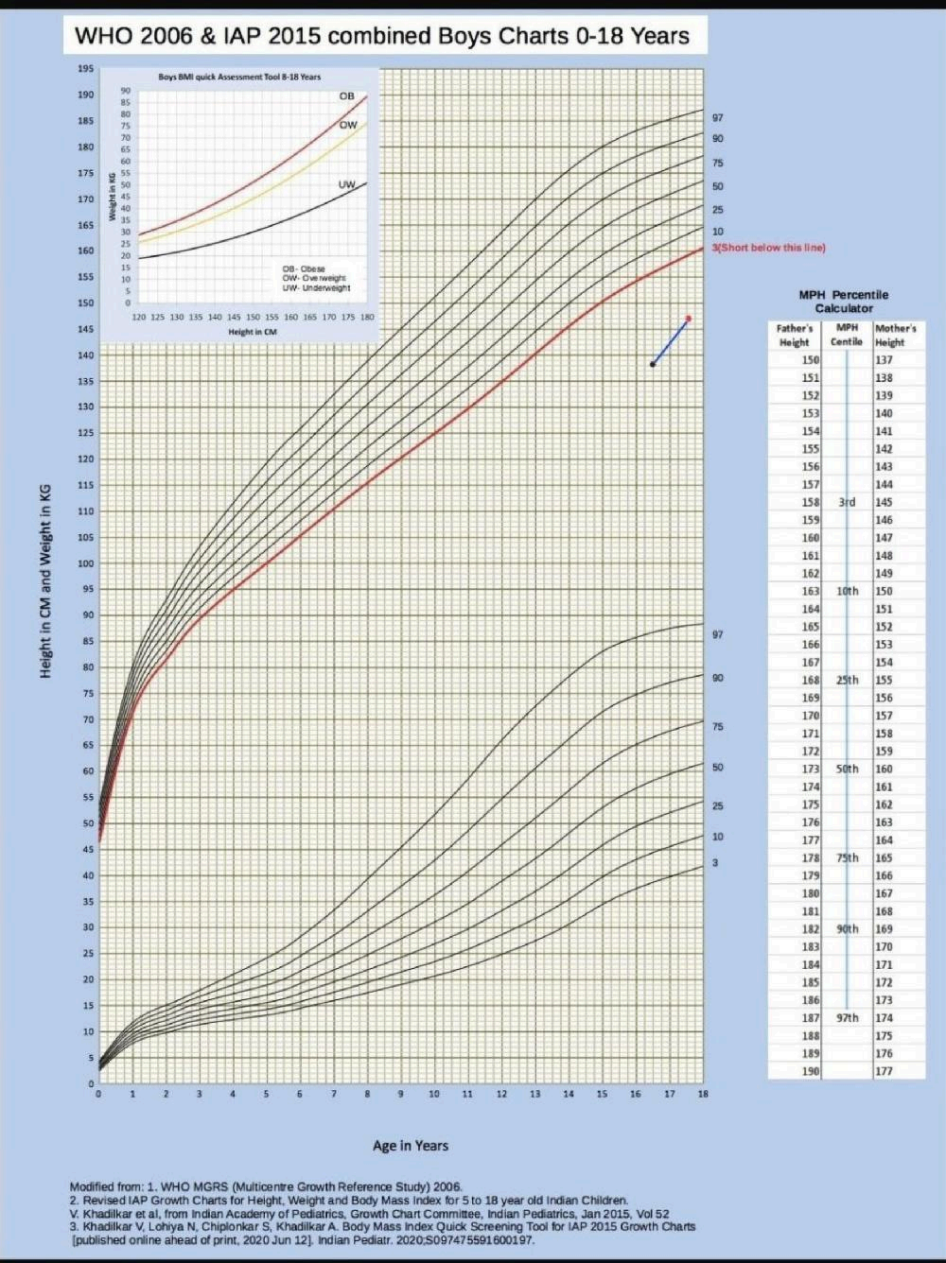
Height representation on growth chart: the black dot and red dot represent height at the time of presentation (138 cm) and height after 1 year of testosterone therapy (147 cm), respectively. Line joining the 2 dots shows the growth trajectory.

At the last follow-up, the patient was 17 years 6 months old. He had been receiving 150 mg intramuscular testosterone injections every 4 weeks. His sexual maturity rating was Tanner pubic hair stage 4. His bilateral testicular volume was still 2 mL. Stretched penile length had increased to 10 cm. His height had increased to 147 cm; his weight was 34 kg, and his bone age was between 14 and 15 years. No adverse effects were encountered following intramuscular testosterone therapy. His linear growth velocity improved after 1 year of testosterone therapy (Fig. 4).

## Discussion

Our patient presented with short stature and a phenotype of nIHH, characterized clinically by delayed puberty, micropenis, and eunuchoid body proportions without impairment of the sense of smell. Gonadotropins and testosterone levels were low-normal, and other pituitary hormones were in the normal range.

Constitutional delay in growth and puberty (CDGP) is a common cause of short stature with delayed puberty. However, our patient exhibited eunuchoid body proportions with stretched penile length well below the range for age at presentation according to normative Indian data [6] and a bilateral testicular volume of only 2 mL at 16 years of age. He failed to enter spontaneous puberty even after 1 year of testosterone therapy, which is less likely for CDGP, where endogenous pubertal progression typically resumes following a short course of sex steroid priming. The lack of spontaneous pubertal progression by 17 years of age even after sex steroid administration, micropenis, low testicular volume, eunuchoid body proportions, and inappropriately low testosterone levels favor the diagnosis of CHH rather than CDGP [7]. There were no clinical or biochemical signs of chronic systemic illness or malabsorption that could otherwise result in functional hypogonadism or pubertal delay.

In our patient, genetic analysis revealed a homozygous single-nucleotide deletion (c.1379delA) in the *NR5A1* gene, not previously reported in the literature. *NR5A1* is a critical transcription factor involved in the development of ventromedial hypothalamus, gonads, and adrenal glands and in the regulation of gonadotroph function in the anterior pituitary. The pathogenic variant of the *NR5A1* gene usually manifests as disorders of sex development in individuals with a 46,XY karyotype, sometimes accompanied by adrenal insufficiency.

A series of in vitro studies has also established the role of *NR5A1* in regulation of the α subunit of glycoprotein hormones, LH β, FSH β, and the GnRH receptor in pituitary gonadotrophs [8].

The *NR5A1(SF-1*) gene mutation linked to hypogonadotropic hypogonadism has been found in knockout mouse. In a *SF-1* knockout mice model, selective SF-1 deletion in the pituitary resulted in a marked decrease in gonadotroph expression and development of hypoplastic gonads, thus highlighting the critical role of SF-1 in pituitary gonadotroph function [9]. Our case represents an example of a variant of *NR5A1* in isolated hypogonadotropic hypogonadism in humans.

A study from China identified variants in *NR5A1* and *PIN1* in children diagnosed with hypogonadotropic hypogonadism. In that study, 5 out of 50 patients had 4 new heterozygous variants in *NR5A1*. Among them, 2 patients had nucleotide substitutions, c.437G > C (p.Gly146Ala) in exon 4; 1 patient had a synonymous variant, c.351C > G (p.Gly117Gly) in exon 4; and the remaining 2 had a *NR5A1* variant in noncoding regions at position 1655 C > T and 2973 T > C in exon 7 [10]. In this study, none of the patients had associated adrenal insufficiency. In 1 patient, there was a homozygous mutation in the coding region of exon 7 of the *NR5A1* gene, which is different from the aforementioned variants, and our patient did not have adrenal insufficiency. In cases where *NR5A1* variants are associated with adrenal insufficiency, the initiation of glucocorticoid therapy is essential. However, this was not applicable in our case as there were no clinical or biochemical features suggestive of adrenal insufficiency.

Genes whose variants have been implicated in nIHH include fibroblast-growth-factor receptor 1 (*FGFR1*), chromodomainhelicase-DNA-binding protein 7 (*CHD 7*), prokineticin 2 (*PROK2*), prokineticin receptor 2 (*PROKR2*), kisspeptin-1 receptor (*KISS1R*), tachykinin precursor 3 (*TAC3*)*, TACR3,* gonadotropin releasing hormone 1 (*GNRH1*), and *GNRHR* [11-16]. However, this homozygous variant in the *NR5A1* gene has not been reported previously in nIHH.

The primary goal of managing CHH in adolescent males is to induce and maintain puberty, support normal physical and psychological development, and ultimately preserve fertility potential when desired. The most commonly used approach for initiating puberty in boys with CHH is intramuscular testosterone therapy. Intramuscular testosterone enanthate or cypionate is typically initiated at a low dose of 50 mg every 4 weeks. The dose is gradually increased every 6 to 12 months up to 250 mg monthly based on the clinical response over a period of 2 to 3 years. Testosterone therapy is monitored clinically through the assessment of pubertal status, growth velocity, skeletal maturation, and development of secondary sexual characteristics and biochemically through evaluation of testosterone levels, hematocrit, and liver function tests. While testosterone therapy is effective for pubertal induction, it does not stimulate testicular growth or spermatogenesis. In patients desiring future fertility or testicular growth, alternative options include gonadotropin therapy with human chorionic gonadotropin in combination with recombinant-human FSH.

Our case highlights the phenotypic heterogeneity associated with the *NR5A1* gene variants. Our report informs about a hitherto unreported variant with homozygous deletion in the *NR5A1* gene in isolated CHH without adrenal insufficiency in a boy. Although the identified variant is interpreted as a variant of uncertain significance, future case reports and further studies may help clarify its pathogenicity and the role of *NR5A1* in the spectrum of CHH.

## Learning Points

- Genetic testing should be considered in CHH as its spectrum is expanding with novel variants in known genes.
- NR5A1 gene variants may manifest without adrenal insufficiency or disorder of sex development.
- To differentiate CHH from CDGP, careful clinical examination, hormonal profiling, and longitudinal follow-up are required.

## Contributors

All authors made individual contributions to authorship. S.S. was involved in drafting and submission of the manuscript. S.G. was involved in drafting and editing the manuscript. N.S. and A.B. were involved in conceptualization, critical review, and management of the patient. R.N. and R.B. were involved in data collection and data analysis. All authors reviewed and approved the final draft.

## Data Availability

Original data generated and analyzed during this study are included in this published article.
